# Incidence and Associated Factors of Failed First Peripheral Intravenous Catheters among Adult Patients at Medical Surgical Wards in Public Referral  Hospitals of West Amhara, Ethiopia, 2021

**DOI:** 10.1155/2022/8261225

**Published:** 2022-01-22

**Authors:** Chanyalew Worku Kassahun, Addisu Taye Abate, Zewdu Baye Tezera, Debrewok Tesgera Beshah, Chilot Desta Agegnehu, Mehmmed Adem Getnet, Hailemichael Kindie Abate, Birhaneslasie Gebeyehu Yazew, Mahlet Temesgen Alemu

**Affiliations:** ^1^Department of Medical Nursing, College of Medicine and Health Sciences, University of Gondar, Gondar, Ethiopia; ^2^Department of Surgical Nursing, College of Medicine and Health Sciences, University of Gondar, Gondar, Ethiopia; ^3^Community Nursing Unit, College of Medicine and Health Sciences, University of Gondar, Gondar, Ethiopia

## Abstract

**Background:**

Complications of peripheral intravenous catheters cause problems in clinical practice and bring high costs in terms of morbidity and mortality of patients. Therefore, this study aimed to assess the incidence and associated factors of failed first peripheral intravenous catheters among adult patients in selected Public Referral Hospitals of West Amhara Regional State, Ethiopia, 2021.

**Materials and Methods:**

An institution-based prospective observational study was conducted among 423 adult patients from January to February 2021. Patients were selected using systematic random sampling techniques. The data were collected using interviewer-based, structured questionnaires and observational checklists. EPI-DATA 3.1 and SPSS version-23 were used for data entry and analysis, respectively. Frequency, percentages, and means were calculated. The outcome variable was determined and graded based on phlebitis and infiltration scales. Binary and multivariable logistics regressions were computed.

**Results:**

Four hundred and seventeen first peripheral cannula sites from 418 patients were followed for 2,565 peripheral catheter hours. A failed first peripheral intravenous catheter was observed in 124 (29.7%, CI: 25.6–34) adult patients. Patients who were female (AOR = 0.4, 95% CI: 0.22–0.74) had cannula duration of 49–72 hours (AOR = 0.31, 95% CI: 0.14–0.7) and 73–96 hours (AOR = 0.39, 95% CI: 0.17–0.9), and patients who had been given electrolytes (AOR = 0.31, 95% CI: 0.11–0.86) were more likely to have failed first peripheral intravenous cannula.

**Conclusions:**

Failed first peripheral intravenous cannula is much higher as compared to the acceptable rate of ≤5% by the Infusion Nurses Society. Hence, all patients with peripheral intravenous catheters are screened for catheter failure at least once a day. Providing appropriate nursing care and patient education is also required to reduce the risks.

## 1. Background

A peripheral intravenous catheter (PIVCs) is a short catheter inserted into the vein on the peripheral areas of the patients [1]. It is the most frequently used invasive clinical/hospital procedure for the patients admitted to the hospital [2–5]. Around 33%–67% of patients have a PIVC inserted during their hospitalization with the cannula remaining in place for considerable duration [6, 7]. It provides access to the administration of intravenous fluids, medications, blood products, and nutrients to the patient [4].

However, the procedure is not free of risks as it puts the patient susceptible to local and systemic complications [4, 8]. It has unacceptably high failure rates [9, 10], and the failure rate lies between 35% and 50% [5]. PIVCs usually fail prematurely before the end of the treatment because of complications [11]. Failures take in the form of phlebitis, infiltration, occlusion/mechanical failure, dislodgment, and infection, any of which alone or in combination leads to removal of the catheter before the end of its intended dwell time [5, 6]. These complications are serious yet preventable adverse events to the patients [12]. They lead to problems in clinical practice and cause patient discomfort/pain, catheter replacement, increased medical treatment and length of hospital stay, with high cost in terms of morbidity and mortality [4, 6, 12], and increases the workload for replacement and follow-up of the devices [11].

In a prospective study on the incidence of PIVC failure at Manacor hospital, Spain was 41.8% [12]. In developing countries, due to scarcity of resources, and economic problems, the incidence of PIVC complications including phlebitis is high [7]. The phlebitis rate ranges from 2.3%–67% [13] and becomes one of the most attention-drawn complications [1, 14]. In another prospective study among adult patients, the incidence of phlebitis was 31.4% [15].

Many factors influenced the development of PIVC complications such as the use of irritant drugs and fluids, size of cannula, anatomical location/site of insertion, large gauge catheter size, duration of cannulation/catheter used for longer than 48 hours, age, gender, and associated diseases [3, 4, 9, 13, 15].

Previous observational studies have recommended interventions namely regular assessment [11] and routine replacement of the catheter as an intervention to reduce the incidence [8, 16]. In addition, the association for Vascular Access (AVA) developed guidelines to ensure a higher level of safety for the patient and promote complication-free device longevity [2]. Jackson recommended points to reduce the incidence of infusion-related complications. These recommendations are observing the site at least daily, securing cannula with a proven dressing, replacing loose, removing contaminated dressings, inserted away from the joints whenever possible, using aseptic technique, considering policy position on re-siting of the cannula, plan, and document continuing care, using the smallest and suitable gauge cannula, and replacing the cannula at the first indication of complications [17].

To date, the issue of PIVC remains unresolved and a public health challenge [18], and the incidence of complications is still higher. There is certainly a pressing need for credible research and forwarding suggestions on this area. However, in Ethiopia, there is no published scientific research describing the incidence of peripheral intravenous catheters' complications among adult patients. The primary aim of this study is to assess the incidence and factors of failed first peripheral intravenous catheters among adult patients in Public Referral Hospitals of Amhara regional state, Ethiopia. The finding would provide directions for the prevention of peripheral catheter failure, improvement of patient outcomes, and reduction of hospital care costs. It would also help operational-level hospitals' managers, Amhara National Regional State Health Bureau, and Federal Ministry of Health Ethiopia to plan such interventions and policy-making, aiming improvement of PIVC.

## 2. Materials and Methods

### 2.1. Study Settings and Period

This study was conducted in Public Referral Hospitals of West Amhara Regional State, Ethiopia from January to February 2021. The regional state contains 28 million population in mid-2018 and it has 14 zones, three city administrations, and 180 woredas (139 rural and 41 urban [19]. It also has 80 hospitals (8 referrals, 2 general, and 73 primaries), 847 health centers, and 3,342 health posts [20, 21]. Despite the increased number of health facilities, shortages of skilled health personnel, medical equipment, drugs, and medical supplies, and inefficient and inequitable use of health resources are the challenges of the region [21, 22]. Five referral hospitals (Debre Markos referral hospital, Tibebe Gion referral hospital, Felege Hiwot referral Hospital, Debre Tabor Referral Hospital, and University of Gondar referral Hospital) were included in the study [21].

### 2.2. Study Design and Population

An institution-based prospective multi-center observational study was conducted among adult patients who were admitted to medical and surgical wards. The source population was all adult patients admitted to medical and surgical wards in each hospital. All adult patients with age greater than or equal to 18 years, who had peripheral intravenous catheter cannula insertions during the time of the study, and agreed to participate in the study were included. Patients with preexisting skin rashes, lacerations, and trauma at insertion sites, unconscious, psychiatric illness, patients who had a history of allergy to any medications, burn at PIVC insertion sites, patients who refused to give written informed consent, and those with previously inserted peripheral intravenous catheter cannula from outside hospitals were excluded [21].

### 2.3. Sample Size, Sampling Technique, and Procedures

To calculate the sample size, we considered the incidence of the first PIVC complication as 50% and with an alpha error of 5% and a power of 95%. Then, 423 sample sizes were required for the study. There are five referral hospitals in West Amhara regional state from which the samples were taken. The total sample size was allocated to each hospital proportionally based on the number of patients they have. Then, patients were selected using systematic random from each hospital [21].

### 2.4. Study Variables

In this study, the dependent variable was failed the first intravenous catheter. Patient-related characteristics such as age, sex; comorbidities such as diabetes, renal problems, liver dysfunctions, surgery, and others; and peripheral intravenous cannula characteristics such as the size of catheter, type of dressing, site of insertion (upper arm, cubital fossa, forearm, wrist, hand), and nature of peripheral intravenous cannula infusate, antibiotics, blood products, and electrolytes were the explanatory variables.

### 2.5. Operational Definition

**PIVC failure** is defined as unplanned PIVC removal with mechanical complications (phlebitis and infiltration) or infection before the completion of any scheduled intravenous therapy [12].
**Failed first intravenous catheter**: A composite measure of first intravenous catheter failure outcomes from phlebitis and infiltration.
  - **Phlebitis** is defined as the presence of two or more signs of pain, tenderness, warmth, erythema, swelling, or a palpable cord with or without purulent drainage from the catheter insertion site. The severity of phlebitis was graded using the Visual Inspection Phlebitis (VIP) scale. The scale can range from 0, indicating no symptoms of phlebitis, to 5, with signs of purulent drainage, redness, and a palpable cord greater than 3 inches. According to the standards of the Infusion Nurses Society (INS), the accepted phlebitis rate is 5% or less [13].
  - **Infiltration** is defined as the permeation of IV fluid into the interstitial compartment, causing swelling of the tissue around the site of the catheter. All PIVCs were changed for a score of 2 or more, determined by the presence of a cold or warm skin region around the insertion site, pain, redness, and/or edema extending from 1 inch to 2 inches from the PIVC site/beyond the tip of the catheter.

### 2.6. Data Collection Tools, Measurements, and Procedures

Interviewer-based structured questionnaires were adapted from validated and standardized existing tools to measure peripheral intravenous catheter-induced complications. The PIVC insertion technique at hospitals is standardized based on the hospital policy and infection control protocol for procedures. But the recommended changing and replacing peripheral intravenous cannula is after 72–96 hours [8]. Observational checklists were used to collect patient-related and peripheral intravenous cannula-related characteristics. The Visual Inspection Phlebitis (VIP) scale [17] from the third edition of the standards for infusion therapy was used to assess phlebitis. The Infusion Nurses Society Infiltration Scale was used to assess infiltration. Only the first insertions of PIVCs were included in the study (21).

### 2.7. Follow-Up of Patients

Recruitment of patients took place at medical and surgical wards immediately at admission. Primarily, data collectors invited the participants to participate in the study. Those who accepted the invitation were asked for their consent. Then identification code was given for records of enrolled participants and follow-up was carried out until the intravenous cannula was discontinued. Data collectors assessed the PIVC site every 12 hours.

### 2.8. Peripheral Intravenous Catheter Care in the Hospitals

PIVCs were usually inserted, and maintained by ward nurses using an aseptic technique. There is no firm policy of scheduled replacement of peripheral catheters during the study period. As a result, most of the peripheral catheters were left in place beyond 72 hours unless a complication is seen. PIVCs that had to be discontinued before 72 hours were because of complications, early discontinuation of IV infusions, and patients' discharge. But the recommended optimal replacement time of an intravenous catheter ranges from when “only clinically indicated” to three days [16, 18].

### 2.9. Data Management and Analysis

EPI- DATA 3.1 and SPSS version-23 software were for data entry and analysis, respectively. Descriptive statistics such as frequency, percentages, means, and standard deviations were calculated. The outcome variable was categorized as having and not having complications (Yes/No) and graded based on phlebitis and infiltration scales. Binary and multivariable logistics regressions analyses were computed. Finally, texts, tables, and graphs were used to report findings.

### 2.10. Quality Assurance Mechanisms

Before collecting the data, the face and content validity of the data collection tool was assured and checked by inviting experts in the field. The data collectors and supervisors were trained about the study purpose and protocol. The research data collection tool was piloted (pre-tested) to check the fitness of the tool for the study settings and necessary correction was made. The investigators exchanged all the necessary information regarding the data collection procedures with the supervisors on a daily basis. Furthermore, the respondents had been given brief orientation (information sheet was read) before the interview, and supervision was also done at the spot by the supervisors. In addition, detailed feedback was provided to the data collectors. The collected data were coded per operational definitions of the study variables and cheeked-rechecked by the principal investigators for its completeness [21].

### 2.11. Ethical Considerations

The overall study protocol was approved by the Institutional Ethical Review Board of the University of Gondar (**December 15**^**th**^**, RNo: V/P/RCS/05/SAS/2020**). Supportive letters were obtained from Amhara Regional state health bureau and then their copies were delivered to each hospital. Oral consent was obtained from each conscious participant and assent from the unconscious, disoriented, and intubated patients after clearly informing the purpose of the study. Name and other personal identifiers were not to be recorded to maintain the privacy and confidentiality of the data. The chance to ask anything about the study as well as the right to refuse or stop respond at any moment was given to participants (21).

## 3. Results and Discussion

### 3.1. Results

#### 3.1.1. Sociodemographic Characteristics of Patients

Of 423 study participants, 418 patients responded to the questions fully, which gave a response rate of 98.8%. A higher proportion of the study participants, 199 (47.6%), were from the University of Gondar Comprehensive Referral Hospital. The age of the patients ranged from 18–90 years (mean 42.85 ± 16.64). In terms of sex and marital status, most of the participants were males (249—59.6%) and married (271—64.8%), respectively. The higher proportion of the participants (105–25.1%) were not able to read and write, and 112 (26.8%) were farmers in occupation (Table 1).

#### 3.1.2. Peripheral Intravenous Cannula-Related Features

Four hundred and seventeen peripheral cannula sites from 418 patients were followed for 2,565 catheter hours. One hundred and twenty-five (29.9%) of patients had associated disease in addition to the main admission diagnosis.

Majority of peripheral catheter sizes were G-18 in 218 (52.2%) and G-20 in 183 (43.8%), respectively. Plaster-based dressing was applied for the majority of catheter sites, 415 (99.3%). 166 PIVCs were frequently inserted in forearms (39.7%) followed by wrists in 107 (25.6%). Antibiotics were the major infusate, 376 (90%) administered through the peripheral cannula followed by glucose, 183 (43.3%). The duration of the peripheral cannula on the patients ranged from an hour to 168 hours. A larger proportion (224—53.6%) of the cannula stayed with the patient for less than 48 hours and 49 (11.7%) of the cannula stayed for more than 96 hours. (Table 2).

#### 3.1.3. The Incidence and Severity of Peripheral Cannula-Related Complications

Complication related to the peripheral catheter was observed in 124 (29.7%, CI: 25.6–34) patients. Phlebitis accounted for the majority, 100 (23.9%) of the complications. Regarding the severity of the complications, the majority of the complication was grade 1 in both phlebitis and infiltration (Table 3).

#### 3.1.4. Factors Associated with Peripheral Cannula Complications

Bivariate and multivariable logistic regression analysis was carried out to see the effect of independent variables on the dependent variable. In the bivariate analysis age category from 34–50 years, being female sex, presence of comorbidity, peripheral cannula duration more than 24 hours, and administering electrolytes were significant factors for the incidence of PIVC complications. The patients who were in the age group of 34–50-year-old were 0.56 times more likely to have PIVC complications as compared to the age group ≥50 years old (COR = 0.56, 95% CI: 0.33–0.96). The patients who had comorbidity were 0.56 times more likely to have PIVC complications as compared to those who had not (COR = 0.56, 95% CI: 0.36–0.88).

Being female sex, peripheral cannula duration of 48–96 hours, and administering electrolytes were significant factors for the incidence of PIVC complications in the multivariable logistic regression analysis. Being female patients were 0.4 more likely to have PIVC complications as compared to male patients (AOR = 0.4, 95% CI: 0.22–0.74). Patients who had peripheral cannula duration from 49–72 hours were 0.31 more likely to have PIVC complications as compared to patients who had less than 24 hours duration (AOR = 0.31, 95% CI: 0.14–0.7). Patients who had peripheral cannula duration from 73–96 hours were 0.39 more likely to have PIVC complications as compared to patients who had less than 24 hours duration (AOR = 0.39, 95% CI:0.17–0.9). Patients who had been given electrolytes through the PIVC were 0.31 more likely to have PIVC complications than their counterparts (AOR = 0.31, 95% CI: 0.11–0.86) (Table 4).

### 3.2. Discussion

In this study, a complication related to the peripheral catheter was observed in one-third of the patients, and phlebitis accounted for the majority of the complications. The majority of the complication was grade −1 in both phlebitis and infiltration. Age category from 34–50 years old, being female sex, presence of comorbidities, peripheral cannula duration more than 24 hours, and administering electrolytes were significant factors for the incidence of a failed first peripheral intravenous catheter.

This study revealed that one-third of the first peripheral intravenous cannula had catheter failure outcomes either in the form of phlebitis or infiltration. This rate is consistent with the study conducted in Air Force Hospital Kalaikunda [15], and the University of Tokyo in Japan (29.2%) [23]. But it is much higher than the expected rate (≤5%) of PIVC complications recommended by the Infusion Nurses Society [13]. This might be due to scarcity of resources and economic problems to change the PIVC as per the recommendations [7]. It is also higher than in another study conducted in Japan (7.5%) ([14]. This might be because study participants were from intensive care units in Japan where serious follow-up of the patients had taken place. It is lower than a study finding at King Abdulaziz Medical City, Riyadh, Saudi Arabia (39.3%) [24]. The difference might be since, in this study, the incidence of failed PIVC was calculated from the first inserted peripheral cannula and composited from phlebitis and infiltration, but it was calculated from the whole inserted peripheral cannula and other complications were added in Saudi Arabia.

The current study indicated that the majority of the complications were grade −1 in both phlebitis and infiltration. This finding is in line with the study finding in China where most of the complications in phlebitis (88.4%) and infiltration (93.7%) were grade −1(10). It is also supported by a study conducted in Japan where 72.6% of phlebitis was Grade −1 [14].

The patients' characteristics and condition are also risk factors for phlebitis that should be considered in an individual's care [24]. The current study indicated that the odds of having failed PIVC are higher among female patients as compared to male patients. This finding is supported by a study conducted at Queensland, Australia where female sex was a non-modifiable factor associated with an increased risk of PIVC failure [6]. This finding was in agreement with the conclusion of a study finding at King Abdulaziz Medical City, Riyadh, Saudi Arabia where phlebitis was predicted with female sex [24].

This study reported that patients who had peripheral cannula duration of 48–96 hours were more likely to have PIVC complications as compared to patients who had less than 24 hours duration. According to the Infusion Nurses Society (INS) guidelines, there is no need to change the peripheral intravenous cannula for adult patients after 72 hours unless clinically indicated [16].

The Centers for Disease Control and Prevention advocate replacing catheters every 72–96 hours in the adult patients in order to reduce complications [8, 24]. However, in the present study peripheral catheters left in for over 96 hours did not show a higher incidence of complications. This finding was in line with a study's findings in China and King Abdulaziz Medical City, Riyadh, Saudi Arabia where duration for PIVCs insertion was not a significant predictor of the complications [10]. This was in agreement with the notion that routine replacement of PIVCs does not affect on the incidence of catheter failures [24] and the catheter for adult patients should be changed when clinically indicated only [16]. But, breaching the recommendation of routine removal of catheters at 72–96 hours is up to the decision to the clinical judgment of nurses [12].

#### 3.2.1. Strength and Limitation of the Study

A prospective follow-up study was employed to see the development of the outcome variable, and all efforts were made to avoid sources of bias related to patient follow-up during the study. As a result, the finding of this study can be reasonably generalized to a larger group of population and institutions.

The interpretation of this finding should account for the following limitations. First, the follow-up time of the study was short. Second, the outcome occurrence after removal of PIVCs and discharging of the patient was not followed.

## 4. Conclusions

The incidence of the failed first peripheral intravenous catheter was much higher than the acceptable rates. A large proportion of first intravenous catheter failure occurred in the form of Phlebitis. Comorbidity, being female, cannula duration of more than 48 hours, and type of infusate, electrolytes were the factors associated with PIVC failure.

Hence, all patients with peripheral intravenous catheters should be screened for complications of the PIVC at least once daily as recommended by the CDC guideline [25]. It is also important to have appropriate nursing care and patient education to reduce the risk factors. An observation chart to document the development of signs of PIVC complications may be developed in hospitals. This would help detect PIVC complications much earlier.

### 4.1. Clinical Implication of the Study

As shown in the result section of this study and the literature reviewed, understanding the rate and factors of failed peripheral intravenous catheters is important to fill the gap and strengthen the quality of patient care. The result of the current study reflects the finding may help to guide health care workers working in institutions with facilities equal to the center in which the study was conducted.

## Figures and Tables

**Table 1:** Sociodemographic characteristics of participants to assess the incidence and associated factors of failed first intravenous catheter among adult inpatients at medical-surgical wards in Public Referral Hospitals of West Amhara Regional State, Ethiopia, 2021 (*N* = 418).

Variable category		Frequency	Percentage
Study hospitals	University of Gondar comprehensive referral hospital	199	47.6
	Tebebe Gion referral hospital	60	14.4
	Felegehiwot referral hospital	55	13.2
	Debretabor referral hospital	54	12.9
	Debremarkos referral hospital	50	12.0

Patient unit category	Medical ward	197	47.1
	Surgical ward	221	52.9

Age category	18–33	147	35.2
	34–50	146	34.9
	>50	125	29.9

Sex	Male	249	59.6
	Female	169	40.4

Marital status	Single	86	20.6
	Married	271	64.8
	Divorced	38	9.1
	Widowed	23	5.5

Educational level of patient	Not able to read and write	105	25.1
	Able to read and write	82	19.6
	Grade 1–4	20	4.8
	Grade 5–8	50	12.0
	Grade 9–12	80	19.1
	Colleague and above	81	19.4

Occupation of patients	Housewife	55	13.2
	Student	41	9.8
	Merchant	69	16.5
	Farmer	112	26.8
	Government employee	78	18.7
	Private employee	37	8.9
	Daily borer	21	5.0
	Other specify^*∗*^	5	1.2

^*∗*^No job = 1, priest = 1, and retired = 3.

**Table 2:** Peripheral intravenous cannula-related features to assess the incidence and associated factors of failed first intravenous catheter among adult inpatients at medical-surgical wards in Public Referral Hospitals of West Amhara Regional State, Ethiopia, 2021 (*N* = 418).

Variable category		Frequency	Percentage
Presence of comorbidity	Yes	125	29.9
	No	293	70.1

Types of comorbidities	Diabetes	26	6.2
	Renal problems	19	4.5
	Liver dysfunctions	5	1.2
	Surgery	5	1.2
	Others^*∗∗∗∗*^	67	16.0

Peripheral intravenous cannula-related characteristics			
Size of cannula	G16	9	2.2
	G18	218	52.2
	G20	183	43.8
	G22	8	1.9

Type of dressing	Plaster	415	99.3
	Transparent	2	0.5
	Gauze	1	0.2

Site of insertion	Upper arm	22	5.3
	Cubital fossa	31	7.4
	Forearm	166	39.7
	Wrist	107	25.6
	Hand	90	21.5
	Others^∗∗^	2	0.5

Duration of the peripheral cannula	<24 hours	122	29.2
	24–48 hours	102	24.4
	49–72 hours	83	19.9
	73–96 hours	61	14.6
	>96 hours	49	11.7

Nature of peripheral intravenous cannula infusate	Glucose (0.09%/0.45% NaCl, Dextro/NaCl)	183	43.3
	Antibiotics	376	90
	Glucose (D30 water)	59	14.1
	Blood products	31	7.4
	Electrolytes	32	7.7
	Others ^∗∗∗^	56	

^∗∗^: Jugular vien-1, Leg = 1. ^∗∗∗^Antihypertensive, antimalaria, antipain, diuretics, atropine, PUD medications. ^∗∗∗∗^Anemia, aspirational pneumonia, asthma, bed sore, BPH, CAP, dermatitis, disseminated TB, fracture, gastric cancer, HAP, hemorrhoid, PCP, RVI, pancytopenia, paraplegia, PTB, seizure, and leishmania.

**Table 3:** The incidence and severity of peripheral cannula-related complications to assess the incidence and associated factors of failed first intravenous catheter among adult inpatients at medical-surgical wards in Public Referral Hospitals of West Amhara Regional State, Ethiopia, 2021 (*N* = 418).

Variable category		Frequency	Percentage
Presences of complication related to the cannula	Yes	124	29.7
	No	294	70.3

Types of complication			
Phlebitis	Yes	100	23.9
	No	318	76.1

Grading of phlebitis	Grade 1	69	16.5
	Grade 2	29	6.9
	Grade 3	2	.5

Infiltration	Yes	25	6
	No	393	94

Grading of infiltration	Grade 1	19	4.5
	Grade 2	4	1.0
	Grade 3	2	.5

**Table 4:** Factors associated with the incidence of failed first intravenous catheter among adult inpatients at medical-surgical wards in Public Referral Hospitals of West Amhara Regional State, Ethiopia, 2021 (*N* = 418).

Variable		Is there a complication related to the peripheral cannula		COR (95% CI)	AOR (95% CI)
		Yes	No		
Patient unit category	Medical ward	64	133	**1**	1
	Surgical ward	60	161	1.29(0.85–2)	0.99(0.53–1.86)

Age category	18–33	44	103	0.71(0.41–1.22)	1.05(0.47–3.36)
	34–50	51	95	**0.56(0.33–0.96)** ^*∗*^	0.79(0.39–1.62)
	>50	29	96	**1**	**1**

Sex	Male	56	193	**1**	**1**
	Female	68	101	**2.32(1.51–3.56)** ^∗∗^	**0.4(0.22–0.74)** ^∗∗^

Marital Status	Single	22	64	1.87(0.71–4.92)	2.5(0.0.65–9.76)
	Married	80	191	1.54(0.64–3.69)	1.35(0.43–4.23)
	Divorced	13	25	1.24(0.42–3.61)	1.9(0.46–7.8)
	Widowed	9	14	**1**	**1**

Educational level of patient	Not able to read and write	26	79	1.6(0.85–3.04)	1.4(0.45–4.47)
	Able to read and write	21	61	1.54(0.78–3.01)	0.96(0.33–2.85)
	Grade 1–4	4	16	2.11(0.64–6.93)	1.86(0.36–9.5)
	Grade 5–8	13	37	1.5(0.69–3.28)	1.38(0.49–3.89)
	Grade 9–12	32	48	0.79(0.42–1.5)	0.6(0.25–1.52)
	Colleague and above	28	53	**1**	**1**

Occupation of patients	House wife	17	38	**1**	
	Student	14	27	0.56(0.06–5.38)	0.37(0.1–1.4)
	Merchant	18	51	0.48(0.05–4.74)	1(0.35–2.86)
	Farmer	28	84	0.71(0.07–6.76)	0.92(0.36–2.32)
	Government employee	25	53	0.75(0.08–6.99)	0.433(0.13–1.48)
	Private employee	15	22	0.53(0.06–4.99)	0.53(0.16–1.75)
	Daily borer	6	15	0.37(0.04–3.6)	0.48(0.13–1.74)
	Other specify^*∗*^	1	4	0.63(0.06–6.8)	0.85(0.07–9.6)

Presence of comorbidity	Yes	48	77	**0.56(0.36-0.88)** ^*∗*^	0.87(0.48–1.59)
	No	76	217	1	**1**

Size of cannula	G16-G18	59	168	0.68(0.45–1.04)	1.74(0.92–3.13)
	G20-G22	65	126	1	**1**

Site of insertion	Upper arm	7	15	**1**	**1**
	Cubital fossa	10	21	0.98(0.3–3.2)	2.27(0.46–11.3)
	Forearm	51	115	1.1(0.41–2.74)	1.78(0.47–6.64)
	Wrist	31	76	1.14(0.43–3.1)	2(0.52–7.78)
	Hand	23	67	1.4(0.5–3.75)	1.35(0.35–5.27)
	Others^∗∗^	2	0		

Peripheral cannula duration	<24 hours	21	101	1	**1**
	24–48 hours	34	68	**0.42(0.22–0.78** ^*∗*^	0.58(0.28–1.2)
	49–72 hours	35	48	**0.29(0.15–0.54)** ^∗∗^	**0.31(0.14–0.7)** ^∗∗^
	73–96 hours	21	40	**0.4(0.2–0.8)** ^*∗*^	**0.39(0.17–0.9)** ^*∗*^
	>96 hours	13	36	0.58(0.26–1.27)	0.57(0.23–1.4
Nature of peripheral intravenous cannula infusate					
Glucose (0.09%/0.45% NaCl, Dextro/NaCl)	Yes	14	23	0.8(0.53–1.23)	0.89(0.51–1.54)
	No	22	51	1	1

Antibiotics	Yes	34	70	0.83 (0.4–1.7)	1.16(0.49–2.75)
	No	2	4	1	1

Glucose (D30 water)	Yes	6	13	0.82(0.45–1.5)	1.79(0.68–4.7)
	No	30	61	1	1

Blood products	Yes	5	6	0.52(0.25–1.1)	0.6(0.23–1.54)
	No	31	68	1	1

Electrolytes	Yes	3	4	**0.37(0.18–0.76)** ^*∗*^	**0.31(0.11–0.86)** ^*∗*^
	No	33	70	1	

^*∗*^Significant at *P* < 0.05; ^∗∗^highly significant at *P* < 0.01. The bold variables are significantly associated in both bi-variable and multivariate analyses.

## Data Availability

The data set generated in this study will be available upon reasonable request from the corresponding author.

## Abbreviations

AOR: Adjusted odds ratio
AVA: Vascular access
BPH: Benign prostate hyperplasia
CAP: Community-acquired pneumonia
CI: Confidence interval
COR: Crude odds ratio
HAP: Hospital-acquired pneumonia
INS: Infusion Nurses Society
IV: Intravenous
PCP: Pneumocystis cairn pneumonia
PIVC: Peripheral intravenous catheter
PTB: Pulmonary tuberculosis
PUD: Peptic ulcer disease
RVI: Retroviral infection
SPSS: Statistical Package for Social Sciences
TB: Tuberculosis.
